# Current Advances and Limitations in Modeling ALS/FTD in a Dish Using Induced Pluripotent Stem Cells

**DOI:** 10.3389/fnins.2017.00671

**Published:** 2017-12-13

**Authors:** Wenting Guo, Laura Fumagalli, Robert Prior, Ludo Van Den Bosch

**Affiliations:** ^1^KU Leuven-Department of Neurosciences, Experimental Neurology and Leuven Institute for Neuroscience and Disease, Leuven, Belgium; ^2^Laboratory of Neurobiology, VIB & KU Leuven Center for Brain & Disease Research, Leuven, Belgium

**Keywords:** amyotrophic lateral sclerosis, frontotemporal dementia, iPSC, neurodegeneration, motor neuron

## Abstract

Amyotrophic lateral sclerosis (ALS) and frontotemporal dementia (FTD) are two age-dependent multifactorial neurodegenerative disorders, which are typically characterized by the selective death of motor neurons and cerebral cortex neurons, respectively. These two diseases share many clinical, genetic and pathological aspects. During the past decade, cell reprogramming technologies enabled researchers to generate human induced pluripotent stem cells (iPSCs) from somatic cells. This resulted in the unique opportunity to obtain specific neuronal and non-neuronal cell types from patients which could be used for basic research. Moreover, these *in vitro* models can mimic not only the familial forms of ALS/FTD, but also sporadic cases without known genetic cause. At present, there have been extensive technical advances in the generation of iPSCs, as well as in the differentiation procedures to obtain iPSC-derived motor neurons, cortical neurons and non-neuronal cells. The major challenge at this moment is to determine whether these iPSC-derived cells show relevant phenotypes that recapitulate complex diseases. In this review, we will summarize the work related to iPSC models of ALS and FTD. In addition, we will discuss potential drawbacks and solutions for establishing more trustworthy iPSC models for both ALS and FTD.

## Introduction

### Amyotrophic lateral sclerosis

Amyotrophic lateral sclerosis (ALS) is a progressiveneurodegenerative disease caused by the selective death of both upper and lower motor neurons (Renton et al., 2014). The disease is also known as Lou Gehrig's disease, as it was named after the American baseball player who was diagnosed with ALS in 1939 and died as a result of the disease (Taylor et al., 2016). Typically, the onset of this disease is in late midlife and it is mostly fatal within 3–5 years after the detection of the first symptoms (Renton et al., 2014; Taylor et al., 2016). Currently, there are no effective treatments for ALS. The first FDA-approved drug for the treatment of ALS was riluzole, which is thought to interfere with glutamate metabolism. However, riluzole only increases survival by a few months (Petrov et al., 2017). Recently, edaravone was approved by FDA after it was shown that intravenous injection of this anti-oxidant slows down the disease progression in a subpopulation of ALS patients (Hardiman and van den Berg, 2017).

The incidence of ALS varies between 0.3 and 3.6 new cases per 100,000 per year depending on the demographics (Henry et al., 2015). In 90% of ALS cases, there is no familial history and these patients are considered as sporadic ALS patients. In ~10% of patients, the disease is inherited and these are classified as familial ALS (Renton et al., 2014; Henry et al., 2015). The genetic etiology of sporadic ALS and of ~30% of familial ALS (European descent) is still unknown (He et al., 2015). The major genetic pattern of inheritance of familial ALS is autosomal dominant, but other hereditary patterns have been reported. The most prevalent mutated genes are *superoxide dismutase 1* (*SOD1*), *chromosome 9 open reading frame 72* (*C9ORF72*), *TAR DNA-binding protein 43* (*TARDBP*) and *fused in sarcoma* (*FUS*) (Renton et al., 2014).

### Frontotemporal dementia

Frontotemporal dementia (FTD) is comprised of a group of disorders caused by progressive neurodegeneration in the frontal and/or temporal lobes of the brain (Bang et al., 2015). It is also called Pick's disease after the physician Arnold Pick, who first described the disease in 1892 (Boxer and Miller, 2005). FTD accounts for up to 10–15% of all dementia cases and is considered the second most common form of early-onset dementia in people younger than 65 years of age, after Alzheimer's disease (Karageorgiou and Miller, 2014; Lashley et al., 2015). Patients show changes in social and personality behavior, apathy, blunting of emotions, and/or deficits in both expressive and receptive language (Bang et al., 2015; Burrell et al., 2016). Unfortunately, there is no effective treatment for FTD yet. The exact cause of FTD is still unknown, although in 40–50% of FTD cases there is a family history (Fong et al., 2012; Ng et al., 2015). Several gene mutations were linked to FTD. The most frequently mutated genes include *Microtubule Associated Protein Tau* (*MAPT*), *Progranulin* (*GRN*), and *C9ORF72* (Bang et al., 2015; Burrell et al., 2016).

### ALS and FTD, the extremities of a disease spectrum

Although ALS and FTD mostly occur as separate diseases, there are more and more common aspects identified for both disorders. Clinically, a number of patients diagnosed with ALS show signs of FTD such as behavioral, cognitive, or language dysfunctions (Ling et al., 2013; Ng et al., 2015). The frequency of FTD clinical features in ALS varies amongst different reports, but it was claimed that some FTD-related symptoms can be detected in up to 50% of ALS patients (Ferrari et al., 2011). Additionally, the opposite also occurs, in that some FTD patients develop motor symptoms during the disease process. Moreover, ALS and FTD can occur within the same family (Ferrari et al., 2011; Ling et al., 2013; Ng et al., 2015). In terms of genetics, hexanucleotide repeat expansions in *C9ORF72*, as well as mutations in the *Valosin-Containing Protein* (*VCP*) gene are found in both ALS and FTD (Ling et al., 2013). Furthermore, the repeat expansions in *C9ORF72* cause the disease in a high proportion of families in which both FTD and ALS occurs (Cooper-Knock et al., 2015). Interestingly, FTD and ALS also share some pathological hallmarks; the most important being the presence of neuronal inclusions of TDP-43, the gene product of the *TARDBP* gene (Ling et al., 2013). Similarly, FUS inclusions are observed in a limited number of ALS, as well as FTD patients (Ling et al., 2013). In addition, nuclear repeat-containing RNA foci as well as inclusions containing dipeptide repeat proteins (DPRs), both specific pathological characteristics of patients with *C9ORF72* expansions, are detected in *post mortem* material of these ALS and FTD patients (Ling et al., 2013; Cooper-Knock et al., 2015).

### Multiple pathological mechanisms have been associated with ALS/FTD

A large number of biological processes have already been suggested to be involved in ALS and some of these could also play a role in FTD. Amongst others, these include excitotoxicity, hyperexcitability, astrocytosis, neuroinflammation, mitochondrial dysfunction, axonal abnormalities, dysregulated autophagy, abnormal RNA metabolism, problems with stress granule dynamics, and nucleocytoplasmic transport defects.

The pathogenic mechanism for which there is ample evidence that it indeed plays a role in ALS patients is excitotoxicity (Van Den Bosch et al., 2006). Excitotoxicity is the process of neuronal degeneration caused by overstimulation of glutamate receptors. One of the major arguments for an involvement of excitotoxicity in the ALS disease process is that riluzole has anti-excitotoxic properties (Miller et al., 2003).

More recently, at least part of the therapeutic effect of riluzole was linked to a (transient) effect on axonal and cortical hyperexcitability (Vucic et al., 2013). Peripheral and central hyperexcitability mirrors clinical features (fasciculations) and neurophysiological findings (increased intracortical excitability). Although there are no treatments approved by the FDA for FTD until now, one transgenic mouse model carrying human TDP-43^A315T^ showed hyperactive somatostatin interneurons that disinhibited pyramidal neurons, which contributed to excitotoxicity (Zhang W. et al., 2016). In addition, another transgenic mouse model with a human Tau^A152T^ mutation showed excitotoxicity mediated by NR2B-containing NMDA receptors due to enhanced extracellular glutamate (Decker et al., 2016).

It is well-known that astrocytes as well as microglia are activated during the disease process. The activated astrocytes release toxic factors, of which the identity is largely unknown. Furthermore, they seem to be soluble in nature, as mutant astrocyte-conditioned medium is also toxic for primary motor neurons of embryonic stem cell (ESCs) derived motor neurons (Nagai et al., 2007). In addition, mutant SOD1 expressing astrocytes fail to protect neurons from excitotoxicity. Astrocytes can increase the excitability and Ca^2+^ influx in motor neurons by increasing the Na^+^ current or by decreasing the neuronal GluR2 expression leading to a higher Ca^2+^ permeability of the AMPA receptor, ultimately leading to neuronal excitotoxicity (Van Damme et al., 2007; Fritz et al., 2013). When microglia are activated during the disease process, the cell body enlarges and the processes get thicker. Microglia mimic properties of antigen-presenting cells and start to interact with T-cells, which infiltrate in the spinal cord and cortex (Engelhardt et al., 1993; Alexianu et al., 2001). This microglial activation starts before disease onset and the number of activated microglia and infiltrated T-cells increases with disease progression (Hall et al., 1998; Alexianu et al., 2001). Similarly, when GRN was injected in mouse brain, an increase of Iba1-positive microglia around the injection site was observed. This indicates that there could be a link between FTD and microglial phagocytosis (Pickford et al., 2011).

Both in animal models and in ALS/FTD patients, dysfunctional mitochondria are observed. Mitochondria are not only crucial for ATP synthesis, but are also involved in intracellular Ca^2+^ homeostasis and induction of apoptosis. As a consequence, abnormal functioning of mitochondria can cause cell death. In the mutant SOD1 mouse model, swollen and vacuolated mitochondria were observed in motor neurons before the first signs of motor neuron degeneration appear (Wong et al., 1995; Kong and Xu, 1998; Higgins et al., 2003). From analysis of the mitochondrial functionality, a decrease of mitochondrial membrane potential was observed in fibroblasts from ALS/FTD patients carrying mutant *TARDBP* (Onesto et al., 2016). Interestingly, increased oxygen consumption and mitochondrial hyperpolarization were observed in fibroblasts from ALS/FTD patients carrying mutant *C9ORF72* (Onesto et al., 2016). Similar abnormalities are found in sporadic ALS/FTD patients (Sasaki and Iwata, 2007). In general, dysfunctions in mitochondrial respiration and ATP synthesis, axonal transport of mitochondria, mitochondrial dynamics, Ca^2+^ buffering (which could lead to excitotoxicity) and induction of apoptosis are all seen in SOD1 mouse models (Kawamata and Manfredi, 2010). Moreover, these abnormalities are not restricted to the mutant SOD1 pathogenesis, also mice overexpressing human TDP-43 have abnormal distribution of mitochondria and changes in mitochondrial dynamics (Xu et al., 2010; Wang et al., 2013), while neuronal cultures expressing ALS-linked FUS mutants contain smaller mitochondria (Tradewell et al., 2012).

Neurons in general and motor neurons in particular are highly dependent on axonal transport mechanisms along their very long axons to bring proteins, organelles and other cargoes to their required sites. Microtubules form tracks along axons on which different cargoes are transported with the help of motor proteins. Since axonal swellings containing neurofilaments were observed in sporadic and familial ALS patients (Okamoto et al., 1990; Sasaki and Maruyama, 1992), axonal transport defects have been intensively studied in ALS (De Vos and Hafezparast, 2017).

ALS is also characterized by the accumulation of ubiquitinated proteins. In the majority of ALS cases, these accumulations contain ubiquitinated TDP-43 even when there are no mutations in the *TARDBP* gene (Neumann et al., 2006). Both autophagy and the ubiquitin-proteasome system (UPS) systems are responsible for the proteostasis in eukaryotes and the prevention of protein aggregation. After deletion of two different essential genes for autophagy (Atg5 or Atg7), each of these transgenic mice develop a neurodegenerative phenotype which suggests an essential role of autophagy in neurons (Hara et al., 2006; Komatsu et al., 2006). Ubiquitin-positive immunoreactive inclusions have been reported as the neuropathological hallmark of FTD patients (Brun et al., 1994).

Disturbances in the RNA metabolism were linked to ALS/FTD after the discovery of mutations in *TARDBP* and *FUS*, encoding two different RNA-binding proteins (TDP-43 and FUS) (Ling et al., 2013). Under normal conditions, both TDP-43 and FUS are localized in the nucleus. In brain and spinal cord of ALS patients, TDP-43 and FUS are in the cytoplasm of the neurons and sometimes also of the glial cells. In response to stress, both TDP-43 and FUS localize to the stress granules present in the cytoplasm. In these stress granules, mRNA is translationally inactive. Both TDP-43 and FUS contain low-complexity domains which are essential for their localization in these stress granules. After periods of stress, the stress granules resolve and mRNA becomes available again to be translated. FUS mutations seem to accelerate the transition from the liquid to the aggregated state in these stress granules (Patel et al., 2015). Interestingly, FUS inclusions have also been reported in FTD patients which do not carry FUS mutations (Lagier-Tourenne et al., 2010). A number of recent papers link the toxicity of the hexanucleotide repeats in *C9ORF72* to an interference with the nucleocytoplasmic transport process (Boeynaems et al., 2016). This could also explain the cytoplasmic mislocalization of TDP-43 in patients with hexanucleotide repeats in *C9ORF72*.

Despite our understanding of the genetic causes and of the pathological processes responsible for ALS/FTD, many potential therapies were unsuccessful in human clinical trials. However, most of these therapeutic strategies were based on preclinical research on animal models. Successful modeling of ALS and FTD by using human materials could deepen our knowledge of these complex diseases, aid in the identification of therapeutic targets and overall, have a significant impact on ALS/FTD research (Picher-Martel et al., 2016; Sances et al., 2016; Lee and Huang, 2017).

## iPSC-derived models for ALS/FTD

Until now, rodent models are the most widely used ALS/FTD models both to study disease mechanisms and to test potential treatments with newly developed compounds (Petrov et al., 2017). With the advancement of genetic and technological approaches, a wide range of additional model systems became available to study these diseases. These model systems differ from *in vitro* to *in vivo*, from invertebrates to vertebrates, from yeast to human iPSCs. It is clear that all these model system have their advantages and disadvantages (Van Damme et al., 2017).

**Figure 1:**
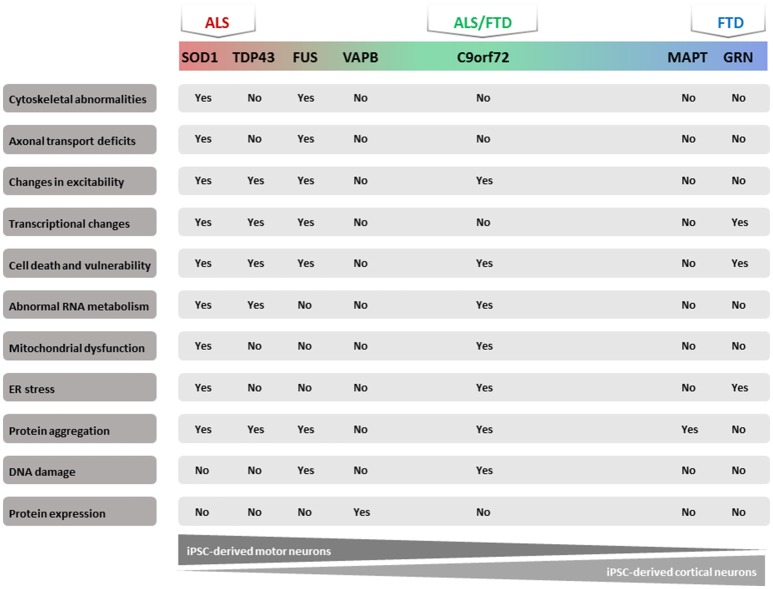
Majorphenotypes in ALS/FTD iPSC models. Summary of observed phenotypes from motor neurons or cortical neurons derived from ALS/FTD patients.

In recent years, several publications identified a number of pathogenic molecular mechanisms of ALS/FTD using human iPSC models (Table 1). Most of these studies focused on familial types of these diseases because the known mutations could give a clue to the underlying mechanism. We will summarize the results obtained with these iPSC models based on the different mutated genes. In addition, we will discuss the results obtained starting from iPSCs from sporadic ALS/FTD patients (Figure 1).

**Table 1:** Summary of published iPSC models of ALS and FTD.

**Disease**	**Gene mutation**	**Reprogramming strategy**	**iPSC-derived cell subtype**	**ALS/FTD phenotype**	**References**
ALS	SOD1^A4V^ SOD1^D90A^	Retroviral/Sendai viral	Motor neurons	Neurofilament misregulation, Neurite degeneration, Mutant SOD1 aggregates	Chen et al., 2014
ALS	SOD1^A4V^	Retroviral	Motor neurons	Survival reduction, Transcriptional change, Mitochondrial defects, Activation of ER stress, Unfolded protein response	Kiskinis et al., 2014
ALS	SOD1^A4V^	Retroviral	Motor neurons	Hyperexcitability, Loss of voltage-gated currents (K^+^)	Wainger et al., 2014
ALS	SOD1^A272C^	Episomal	Motor neurons	Transcriptional changes	Wang et al., 2017
ALS	TDP-43^Q343R^ TDP-43^M337V^ TDP-43^G298S^	Retroviral	Motor neurons	Transcriptional changes, TDP-43 aggregates, Neurite degeneration, Vulnerability to oxidative stress	Egawa et al., 2012
ALS	TDP-43^M337V^	Retroviral	Motor neurons	Survival reduction, TDP-43 aggregates, Vulnerability to PI3K inhibition	Bilican et al., 2012
ALS	TDP-43^M337V^	Retroviral	Astrocytes/Motor neurons	Astrocytes: Survival reduction, Increased insoluble TDP-43, Cytoplasmic mislocalization of TDP-43	Serio et al., 2013
				Motor neuron-astrocyte-coculture: No effect on motor neuron survival	
ALS	TDP-43^A90V^ TDP-43^M337V^	Retroviral	Neurons	Cytoplasmic mislocalization of TDP-43, Decreased miR-9 expression	Zhang et al., 2013
ALS	TDP-43^M337V^	Retroviral	Motor neurons	Hyperexcitability (early stage), Hypoexcitability (late stage), Loss of synaptic activity, Loss of voltage-gated currents (Na^+^)	Devlin et al., 2015
ALS	FUS^M511FS^ FUS^H517Q^	Retroviral	Motor neurons	Hyperexcitability	Wainger et al., 2014
ALS	FUS^R521L^ FUS^R521C^, FUS^R495QfsX527^	Retroviral	Motor neurons	Hypoexcitability, Decreased synaptic activity, Lower sodium to potassium (Na^+^/K^+^) ratios	Naujock et al., 2016
ALS	FUS^P525L^	Episomal	Motor neurons	Cytoplasmic FUS localization, FUS aggregation	Liu et al., 2015
ALS	FUS^H517D^	Episomal	Motor neurons	Cytoplasmic FUS localization, FUS aggregation, Cell death, Aberrant gene expression and splicing	Ichiyanagi et al., 2016
ALS	FUS^R521C^ FUS^R495Qfs527^ FUS^Asp502Thrf^*^27^	Lentiviral	Motor neurons	Cytoplasmic FUS localization, FUS aggregation, Cell death	Higelin et al., 2016
ALS	FUS^R514S^ FUS^R521C^ FUS^P525L^	Lentiviral	Motor neurons	Cytoplasmic FUS localization, FUS aggregation, Cell vulnerability	Lenzi et al., 2015
ALS	FUS^R521H^ FUS^P525L^	Sendai viral	Motor neurons	Cytoplasmic FUS localization, Hypoexcitability, Decreased synaptic activity, Axonal transport defects	Guo et al., 2017
ALS	C9ORF72	Sendai viral	Motor neuorns	Cell death, Abnormal protein aggregation, and stress granule formation, Increased ER stress, Reduced mitochondrial membrane potential, p62 accumulation	Dafinca et al., 2016
ALS	C9ORF72	Episomal	Motor neurons	Cell vulnerability, DNA damage	Lopez-Gonzalez et al., 2016
ALS	C9ORF72	Retroviral	Motor neurons	Hyperexcitability	Wainger et al., 2014
ALS	C9ORF72	Retroviral	Motor neurons	Hyperexcitability (early stage), Hypoexcitability (late stage), Loss of synaptic activity, Loss of voltage-gated currents (K^+^/Na^+^)	Devlin et al., 2015
ALS	C9ORF72	Retroviral	Motor neurons	Transcriptional changes	Kiskinis et al., 2014
ALS	C9ORF72	Retroviral	Neurons	RNA foci, Transcriptional changes, RAN translation products, Vulnerability to glutamate toxicity	Donnelly et al., 2013
ALS	C9ORF72	Episomal	Motor neurons	RNA foci, Transcriptional changes	Sareen et al., 2013
ALS	C9ORF72	Retroviral	Neurons	RNA foci, RAN translation products, Vulnerability to inhibition of autophagy, p62 accumulation	Almeida et al., 2013
ALS	C9ORF72	Not mentioned	Motor neurons	DPRs, Cell-to-cell transmission of DPRs	Westergard et al., 2016
ALS	C9ORF72	Retroviral	Motor neurons	Modulation of actin dynamics	Sivadasan et al., 2016
ALS/FTD	C9ORF72	Lentiviral	iNeurons	Autophagy impairment	Webster et al., 2016
ALS	C9ORF72	Lentiviral/Episomal	Astrocyte/Motor neurons	Abnormal protein aggregation, p62 accumulation	Madill et al., 2017
FTD	C9ORF72	Episomal	Cortical neurons	Nucleocytoplasmic transport defect	Freibaum et al., 2015
ALS	C9ORF72	Retroviral	Neurons	Nucleocytoplasmic transport defect	Zhang et al., 2015
ALS	VAPB^P56S^	Retroviral	Motor neurons	Reduced expression levels of VAPB	Mitne-Neto et al., 2011
ALS	Sporadic ALS	Unknown	Motor neurons	TDP-43 aggregates	Burkhardt et al., 2013
ALS	Sporadic ALS	Lentiviral	Motor neurons	Aberrant gene expression	Alves et al., 2015
ALS	Sporadic ALS	Sendai viral	Astrocytes	Disorganized neurofilaments, Aggregated ubiquitin, Synaptic defects	Qian et al., 2017
FTD	TAU^A152T^	Retroviral	Neurons	Increased tau fragmentation, Increased tau phosphorylation	Fong et al., 2013
FTD	GRN^S116X^	Retroviral	Neurons /Microglia	PGRN haploinsufficiency, Vulnerability to ER stress, Vulnerability to inhibition of proteasome	Almeida et al., 2012
FTD	GRN null	Retroviral	Cortical neurons /Motor neurons	Inefficient cortical neuron formation PGRN haploinsufficiency Transcriptional changes	Raitano et al., 2015

### Advantages of iPSCs as a model system

iPSCs are pluripotent stem cells that are generated directly from adult cells. The iPSC technology was developed by Shinya Yamanaka's lab by introducing four specific transcription factors into adult cells and converting these cells into pluripotent stem cells (Takahashi et al., 2007). As one of the newest ALS model systems, this technology allows for the generation of iPSCs from patient-derived somatic cells from ALS patients and opens new opportunities. Compared with other model systems, there are a number of advantages of the iPSC model system. First, it gives the researcher the opportunity to model ALS with no need to overexpress the transgene with the disease-causing mutation. Second, gene editing technologies, such as CRISPR/Cas9, make it possible to correct these ALS-causing mutations in the same patient-derived iPSCs. This creates an isogenic control with exactly the same genetic background as the patient and makes it also possible to search for disease modifiers in ALS/FTD (Van Damme et al., 2017). Third, the iPSC technology enables researchers to investigate sporadic ALS cases in the laboratory. This has never been possible with other model systems before. Fourth, it allows researchers to investigate human diseases by using patient material to create different cell types. Especially for neurodegenerative diseases, patient-derived neurons are a new research tool which was unavailable before the invention of the iPSC technology. Finally, the constant improvements of differentiation protocols gives the possibility to differentiate iPSCs into many different cell (sub)types (lower motor neurons, upper motor neurons, astrocytes, oligodendrocytes, cortical neurons, …), which allows researchers to investigate the different cell types involved in the disease process or to study the dynamics between the different cell types (Emdad et al., 2012; Maury et al., 2014; Douvaras et al., 2016). The major drawback of this model system is the *in vitro* approach. As a consequence, cross-validation in other *in vivo* systems and/or in patient material remains crucial (Van Damme et al., 2017). Moreover, the costs of the iPSC model system are high compared to some other model systems. However, the valuable output of this relatively new model system is promising and could be very useful in basic research.

### Modeling of ALS in a dish

#### SOD1

In 2014, two back-to-back studies were published which used novel *in vitro* models for ALS by generating iPSC-derived motor neurons from patients carrying a SOD1^A4V^ or a SOD1^D90A^ mutation (Chen et al., 2014; Kiskinis et al., 2014). In each study, the authors used genetic correction of both of these mutations as isogenic controls (Chen et al., 2014; Kiskinis et al., 2014). Both studies reported a high ratio of electrophysiologically active motor neurons differentiated from these iPSCs (Chen et al., 2014; Kiskinis et al., 2014). These motor neurons also recapitulated the spontaneous and progressive decrease in cell viability observed in humans (Chen et al., 2014; Kiskinis et al., 2014). Additionally, ALS-related morphological changes were observed *in vitro*, including a reduction in soma size and an altered dendrite length (Chen et al., 2014). This phenotype was linked to the dysregulation and aggregation of neurofilaments, an event that preceded the occurrence of neuronal apoptosis (Chen et al., 2014). RNA sequencing showed that several genes were misregulated in the patient-derived motor neurons in comparison to isogenic controls. These included transcripts related to cytoskeletal organization, mitochondrial function and structure, and protein translation (Kiskinis et al., 2014). Furthermore, the unfolded protein response was activated (Kaus and Sareen, 2015). Moreover, ER stress occurred in SOD1^A4V^ motor neurons (Kiskinis et al., 2014). In another study, Wainger and colleagues used the same iPSC lines carrying the SOD1^A4V^ mutation and demonstrated that patient-derived motor neurons showed consistent hyperexcitability (Wainger et al., 2014). The authors used this disease-specific phenotype as a tool for drug screening. Retigabine, a clinically approved anticonvulsant, blocked hyperexcitability in these patient-derived motor neurons by activating subthreshold Kv7 currents. Moreover, a significantly increased survival of motor neurons was observed in this study after treatment with retigabine (Wainger et al., 2014). This study provided the rationale to start a clinical trial using retigabine in ALS patients (Noto et al., 2016). However, hypoexcitability in combination with lower Na^+^/K^+^ ratios were observed in motor neurons from an ALS patient carrying the SOD1^D90A^ mutation (Naujock et al., 2016). In addition, this hypoexcitability could be improved by the FDA-approved drug 4-aminopyridine (4AP) which is targeting K^+^ currents (Naujock et al., 2016). Although it is still a debate whether hyper- or hypoexcitability plays a crucial role in ALS, it is evident that electrophysiological changes comprise a major phenotype in iPSC models, which may be a therapeutic avenue worth exploring.

#### TARDBP

All published studies using iPSCs derived motor neurons from mutant *TARDBP* patients reported some significant ALS-related pathological features, including motor neuron degeneration and accumulation of insoluble TDP-43 protein (Egawa et al., 2012).

The research group of Chandran first described a human motor neuron model derived from iPSCs from an ALS patient carrying the TDP-43^M337V^ mutation (Egawa et al., 2012). The functional maturation of motor neurons was not affected by the mutation. However, the survival of these neurons was significantly decreased. Furthermore, these TDP-43^M337V^ motor neurons also showed an increased level of soluble and detergent-resistant TDP-43 compared to healthy control lines. In addition, they also demonstrated that these neurons were more susceptible to inhibition of phosphoinositide 3-kinase (PI3K), while no abnormalities in response to inhibition of mitogen-activated protein kinase (MAPK) or ER stress induction were observed (Egawa et al., 2012). As some neurotropic factors rely on the PI3K pathway, this result supports the importance of neuronal trophic support. In a later publication, this research group used the same iPSC line (TDP-43^M337V^) to generate almost pure astrocytes in order to investigate the astrocyte pathology in ALS (Serio et al., 2013). Astrocytes from TDP-43^M337V^ iPSCs showed cytoplasmic mislocalization of soluble TDP-43 (Serio et al., 2013). In comparison to wild type astrocytes, TDP-43^M337V^ astrocytes showed a cumulative risk of death under basal conditions. However, these cells did not show increased levels of detergent resistant TDP-43, which is different from iPSC-derived motor neurons (Serio et al., 2013). Moreover, the authors used an isogenic control to confirm the observations linking TDP-43 cytoplasmic localization to survival in astrocytes (Serio et al., 2013). In addition, coculturing motor neurons and astrocytes showed that mutant TDP-43 iPSC-derived astrocytes had no effect on motor neuron survival (Serio et al., 2013). Conversely, murine glia overexpressing mutant SOD1 were shown to be toxic to cocultured human iPSC-derived motor neurons (Hedlund and Isacson, 2008).

Egawa and colleagues generated several iPSC lines from three ALS patients with different TDP-43 mutations (Q343R, M337V, and G298S). The authors did not observe that TDP-43 mutations impaired motor neuron maturation (Egawa et al., 2012). However, insoluble TDP-43 inclusions were found in these neurons (Egawa et al., 2012). In contrast to a previous report from Bilican et al. (2012), TDP-43 mRNA levels were higher in mutant cell lines compared to control lines. To explain this discrepancy, the authors suggested that the variation between different iPSC lines and the different purity of motor neurons could account for this difference (Bilican et al., 2012). The study also identified decreased neurite lengths and a higher vulnerability to oxidative stress in the mutant motor neurons. Interestingly, in their drug screening assay, anacardic acid, which is a histone acetyltransferase, was demonstrated as an efficient drug candidate which could reverse the disease-related phenotypes (Egawa et al., 2012).

Serio et al. focused on the physiological aspect of motor neurons carrying the TDP-43^M337V^ mutation by using the same iPSC lines used for generating astrocytes. Hyperexcitability at early stages followed by a progressive loss in action potential output and synaptic activity were shown in the patient-derived motor neurons. As mentioned above, this hyperexcitability phenotype is shared with iPSC-derived motor neurons from patients with SOD1 mutations (Serio et al., 2013).

The group of Gao derived neuronal cultures from iPSCs of an FTD patient, who also showed ALS symptoms, containing an A90V mutation in TDP-43 (Zhang et al., 2013). The A90V mutation is a rare mutation which was identified as a risk factor for both ALS and FTD (Zhang et al., 2013). The disease duration in a patient with this mutation can be more than two decades from the time of diagnosis, which is a particularly long disease duration for ALS. Mislocalized cytoplasmic TDP-43 and decreased expression of total TDP-43 after treatment with staurosporine were observed in the neurons derived from these patient iPSCs (Zhang et al., 2013). Interestingly, the neurons generated from a patient carrying the M337V mutation showed increased TDP-43 aggregates in the absence of any stress (Bilican et al., 2012). This suggested a cell-autonomous toxicity in TDP-43^M337V^ lines. In addition, decreased miR-9 expression was shown in neurons with A90V, as well as with the M337V mutation in TDP-43 (Zhang et al., 2013). Since miRNA misregulation has also been reported in FUS-ALS, this could reveal a common downstream process in different disease subtypes that are caused by mutations in different ALS-associated genes (Bilican et al., 2012; Zhang et al., 2013).

#### FUS

Over the past few years, several FUS-iPSC models have been generated. One publication described motor neuron cultures derived from two FUS-iPSC lines carrying a frameshift mutation at residue 511 (M511FS) or a H517Q point mutation. These motor neurons were shown to be hyperexcitable (Wainger et al., 2014). Conversely, another research group reported hypoexcitability in FUS lines carrying a point mutation (R521L or R521C) and a frame shift mutation (R495QfsX527) (Naujock et al., 2016).

Another study demonstrated cytoplasmic mislocalization and the formation of FUS aggregates in differentiated motor neurons from a patient carrying a P525L mutation (Liu et al., 2015). Similarly, motor neurons derived from ALS patients carrying a H517D mutation in FUS showed cytoplasmic FUS localization and stress granule formation under stress conditions. Moreover, exon array analysis combined with CLIP-seq data revealed aberrant gene expression and/or splicing patterns in patient-derived motor neuron precursor cells (Ichiyanagi et al., 2016). The cytoplasmic FUS mislocalisation was also observed in motor neurons derived from patients with a benign R521C mutation and the more severe R495Qfs527 and Asp502Thrfs^*^27 frameshift mutations (Higelin et al., 2016). Moreover, the amount of cytoplasmic FUS accumulation correlated with the clinical severity of the underlying FUS mutation (Higelin et al., 2016). Furthermore, the severity of the FUS mutation, as well as neuronal aging, also induced spontaneous formation of cytoplasmic FUS inclusions (Higelin et al., 2016). These aggregates showed typical characteristics of FUS-ALS including the presence of methylated FUS (Higelin et al., 2016). Additionally, higher vulnerability and increased DNA damage were observed in patient-derived motor neurons after irradiation (Higelin et al., 2016).

Lenzi et al. used iPSCs derived from patient fibroblasts carrying a R514S or a R521C mutation in combination with iPSC cells, in which the P525L mutation was introduced using transcription activator-like effector nucleases (TALENs)-directed mutagenesis (Lenzi et al., 2015), to differentiate into motor neurons. The presence of mutant FUS resulted in the aberrant localization and recruitment of FUS into stress granules (SGs). However, this only occurred upon induction of stress and the incorporation into SGs was proportional to the amount of cytoplasmic FUS (Lenzi et al., 2015).

We recently generated and characterized iPSCs from ALS patients with R521H and P525L mutations in FUS. Patient-derived motor neurons showed typical cytoplasmic FUS pathology, hypo-excitability, as well as progressive axonal transport defects. Cytoplasmic mislocalization of FUS was most pronounced in the P525L mutant line and was not specific for motor neurons. Both cytoplasmic FUS pathology and axonal transport defects were rescued by “clustered regularly interspaced short palindromic repeats” (CRISPR)/Cas9-mediated genetic correction of the *FUS* mutation in patient-derived iPSCs. Furthermore, we could rescue the defects pharmacologically by histone deacetylase 6 (HDAC6) inhibition and by genetic silencing of HDAC6, which suggests that this could become a new therapeutic strategy for ALS (Guo et al., 2017).

#### C9ORF72

The immense expansion of G_4_C_2_ repeats in *C9ORF72* makes it particularly difficult to develop good animal models. As a consequence, iPSCs could offer the ideal solution for modeling *in vitro* the effect of the hexanucleotide expansion in *C9ORF72*. Three potential mechanisms have been proposed to explain the pathogenic role of the hexanucleotide expansion in *C9ORF72*. First, a reduction in the expression level of *C9ORF72* observed in some *C9ORF72* patients has led to the hypothesis that a loss of *C9ORF72* may contribute to the disease (Waite et al., 2014). Second, the accumulation of RNA foci containing the hexanucleotide expansion found in the brain and spinal cords of patients suggested a disease mechanism, involving a toxic gain of function (DeJesus-Hernandez et al., 2011). This is thought to be mediated by repeat-containing RNA that can potentially bind RNA-binding proteins (DeJesus-Hernandez et al., 2011). Third, both the sense and antisense repeat RNAs can undergo repeat-associated non-ATG (RAN) translation, resulting in the formation of a series of potentially toxic DPRs (Gendron et al., 2013; Zu et al., 2013). RAN translation can occur from both sense and antisense expansion transcripts, resulting in the expression of six RAN proteins (antisense: Pro-Arg, Pro-Ala, Gly-Pro; and sense: Gly-Ala, Gly-Arg, Gly-Pro). These DPRs form neuronal cytoplasmic and intranuclear inclusion in patients carrying the expansion (Gendron et al., 2013; Zu et al., 2013). However, the exact contribution of each of these mechanisms to neuronal death is not yet clear.

Several studies on *C9ORF72* patient iPSCs have shown the potential of iPSCs in recapitulating the major pathological signatures of the disease. In 2013, three groups generated iPSCs from ALS/FTD patients carrying the *C9ORF72* mutation (Almeida et al., 2013; Donnelly et al., 2013; Sareen et al., 2013), in which several aspects of *C9ORF72*-related pathology were observed. RNA foci were detected in patient-derived neurons and the sequestration of RNA binding proteins, including ADARB2, hnRNPA1, and Pur-α, by the expanded RNA repeat was observed (Donnelly et al., 2013; Sareen et al., 2013). Dipeptide RAN pathology was also described in neurons derived from *C9ORF72* patients (Almeida et al., 2013; Donnelly et al., 2013). Interestingly, Westergard and collaborators demonstrated, by using *C9ORF72* iPSCs-derived spinal motor neurons (sMNs), evidence for cell-to-cell spreading of DPRs in a co-culture system of control and *C9ORF72* iPSC-derived sMNs (Westergard et al., 2016). In terms of haploinsufficiency, *C9ORF72* expression was observed to be reduced in patient-derived neurons compared to controls (Almeida et al., 2013; Donnelly et al., 2013), whereas in the study of Sareen et al., no change in *C9ORF72* expression was found in patient-derived motor neurons (Sareen et al., 2013). From a therapeutic point of view, two studies investigated antisense oligonucleotides (ASOs) in *C9ORF72* iPSCs-derived neurons (Donnelly et al., 2013; Sareen et al., 2013). Interestingly, after ASO treatment, the toxicity associated with the endogenous *C9ORF72* mutation was abrogated (Donnelly et al., 2013; Sareen et al., 2013). Moreover, the *C9ORF72* knockdown with ASOs had no impact on iPSCs-derived neuron survival, leading the authors to argue against a loss of function mechanism as the major cause of *C9ORF72* pathology and toxicity seen in iPSCs (Donnelly et al., 2013; Sareen et al., 2013).

Aside from recapitulating the major pathological hallmarks of the disease, iPSC-based platforms have also revealed novel mechanisms of neurodegeneration. Two independent studies showed impaired nucleocytoplasmic transport in patient-derived neurons (Freibaum et al., 2015; Zhang et al., 2015). Zhang and colleagues showed that RanGAP1, a key regulator of nucleocytoplasmic transport, physically interacted with the expanded RNA foci and was mislocalized in the cytoplasm of *C9ORF72* iPSC-derived neurons. In addition, the authors also showed that nuclear import of proteins and RNAs was impaired in patient iPSC-derived neurons, and these deficits were rescued by antisense oligonucleotides targeting the *C9ORF72* RNA (Zhang et al., 2015). Similarly, Freibaum and colleagues observed impaired nucleocytoplasmic transport in *C9ORF72* iPSC-derived neurons. Furthermore, investigation of the total RNA distribution revealed increased nuclear RNA retention in *C9ORF72* iPSC-derived cortical neurons (Freibaum et al., 2015).

Vulnerability to ER and oxidative stress was also investigated in *C9ORF72* iPSCs-derived motor neurons. Dafinca and colleagues showed that *C9ORF72* iPSC-derived motor neurons exhibited loss of Ca^2+^ homeostasis, which was associated with a decrease in mitochondrial potential and an increase in ER stress (Dafinca et al., 2016). Moreover, an increase of yH2AX, a marker of DNA damage, was observed in *C9ORF72* iPSCs-derived motor neurons in an age-dependent manner (Lopez-Gonzalez et al., 2016). Interestingly, the ectopic expression of the DPR protein (GR)_80_, but not (GA)_80_, increased DNA damage in iPSC-derived control neurons. Pharmacological or genetic reduction of oxidative stress partially reduced DNA damage in *C9ORF72*-derived motor neurons suggesting that oxidative stress could play an important role in the disease mechanism (Lopez-Gonzalez et al., 2016).

Several studies performed electrophysiology on *C9ORF72* iPSC-derived neurons to investigate possible impairments in excitability, as observed in *C9ORF72* patients (Williams et al., 2013; Geevasinga et al., 2015). Using multi-electrode array and patch clamp recordings, Wainger and colleagues showed network hyperexcitability in *C9ORF72* iPSC-derived motor neurons (Wainger et al., 2014). In direct contrast, another group showed a diminished capacity to fire continuous spikes upon depolarization in *C9ORF72* iPSC-derived motor neurons compared to control motor neurons (Sareen et al., 2013). Moreover, *C9ORF72* iPSC-derived motor neurons showed altered expression of genes involved in membrane excitability, including the delayed rectifier potassium channel (KCNQ3) which is consistent with hypoexcitability (Sareen et al., 2013). As indicated before, these divergent findings can possibly be explained by the temporal analysis of *C9ORF72* iPSC-derived motor neuron excitability as performed by Devlin and colleagues (Devlin et al., 2015). This research group demonstrated that patient iPSC-derived motor neurons displayed an intrinsic hyperexcitability at early time points (21–28 days in culture), followed by a loss in action potential output and synaptic activity in cells maintained for up to 70 days in culture. This loss of functional output was correlated to a progressive loss of both voltage-activated Na^+^ and K^+^ currents (Devlin et al., 2015). Further experimental evidence is required to determine whether the altered excitability observed in *C9ORF72* motor neurons plays indeed a pathogenic role in ALS or whether it is related to a homeostatic response to specific culture conditions.

Non-cell autonomous toxicity mechanism were also investigated using patient iPSCs-derived cells. Mayer and colleagues showed that *C9ORF72* iPSC-derived astrocytes were toxic when co-cultured with motor neurons. This strongly indicates that astrocytes play an important role in the *C9ORF72*-mediated pathology (Meyer et al., 2014). Similarly, oligodendrocytes from *C9ORF72* patients, obtained through different reprogramming protocols, induced motor neuron death both through conditioned media as well as in co-cultures (Ferraiuolo et al., 2016).

Several studies provided evidence for a role of *C9ORF72* in important cellular functions suggesting that a loss of function of *C9ORF72* could eventually contribute to the disease process. *C9ORF72* is homologous to members of the DENN (differentially expressed in normal and neoplastic cells) domain containing protein family and is predicted to function as a guanine-nucleotide exchange factor for several Rab proteins (Levine et al., 2013). Consistent with the prediction of its function, *C9ORF72* protein was found to colocalize with Rab proteins and to be involved in endosomal trafficking and autophagy (Farg et al., 2014). Subsequently, several studies showed a role of *C9ORF72* in the induction of autophagy (Sellier et al., 2016; Sullivan et al., 2016; Webster et al., 2016; Yang et al., 2016) and reduction in the basal autophagy levels in *C9ORF72* patient-derived neurons (Webster et al., 2016). Moreover, increased level of p62, an autophagy marker, were observed in *C9ORF72* iPSCs-derived neurons (Almeida et al., 2013; Dafinca et al., 2016), reminiscent of the p62 pathology observed in *C9ORF72* patients (Mackenzie et al., 2014). Recently, a study in human iPSC-derived neurons revealed that *C9ORF72* modulates cytoskeletal actin dynamics via phosphorylation of cofilin and shRNA mediated knockdown of *C9ORF72* resulted in axonal outgrowth deficits (Sivadasan et al., 2016).

Taken together, the above studies used iPSC technology to establish *in vitro* models for *C9ORF72*-ALS. Apart from providing novel insights into the disease mechanisms, antisense strategies were suggested as a promising treatment option to counteract the negative impact of the hexanucleotide repeat expansions in *C9ORF72*.

#### VAPB

A mutation in the “*vesicle-associated membrane protein-associated protein B and C” (VAPB*) gene is a rare cause of ALS (subtype 8), and has an autosomal dominant mode of inheritance. Mitne-Neto and colleagues generated iPSCs from an ALS patient with a P56S mutation in *VAPB*, as well as from their non-carrier siblings. These cells could be differentiated into mature motor neuron. No obvious alterations in VAPB distribution were detected in patient-derived cells. Similarly, no aggregates were found in patient cells, even after inducing stress in the cells using the proteasome inhibitor MG132. However, a significant decrease in VAPB protein level was observed in patient-derived motor neurons and the reduction was ~50% compared to controls (Mitne-Neto et al., 2011).

#### Sporadic ALS

Although sporadic forms of ALS account for 90% of total ALS cases, it is much more difficult to model this form of the disease as the exact causes are unknown. As already indicated, iPSC technology could give us the unique opportunity to mimic some pathological and electrophysiological phenotypes *in vitro*.

The first publication on a sporadic ALS iPSC model was from Burkhardt and colleagues. Skin fibroblasts were reprogrammed from 16 sporadic ALS patients, 8 familial ALS patients and 10 healthy persons as controls. Nearly 100 iPSC clones from 34 patients were analyzed. Spontaneous TDP-43 pathology was observed in iPSC-derived motor neurons from 20% of the sporadic ALS patients (Burkhardt et al., 2013). These TDP-43 aggregates were hyperphosphorylated, but no ubiquitination was observed. Additionally, the TDP-43 aggregates in motor neurons differentiated from iPSC were validated in anterior horn neurons of spinal cord and cortical neurons from one of the patients who donated the fibroblasts. Interestingly, this was the first time that a pathological phenotype found in iPSC-motor neurons could be linked to *post mortem* tissue from the same ALS patient, thus validating iPSC-derived models as a powerful tool.

iPSC-motor neurons from one sporadic ALS patient were also used for a drug screening assay (Burkhardt et al., 2013). In total, 1757 bioactive compounds were tested on these neurons and the percentage of neurons containing TDP-43 aggregates was used as the criterion to analyze the efficiency of these compounds. After two rounds of selection, four classes of compounds, including cyclin-dependent kinase inhibitors, Digoxin, Lanatoside C, and Proscillaridin A, were obtained that reduced in a dose-dependent manner the percentage of cells with TDP-43 aggregates (Burkhardt et al., 2013). These findings rely on a prominent role of protein aggregation in the progression of neuronal degeneration in ALS (Burkhardt et al., 2013). Another large gene profiling experiment starting from differentiated motor neurons from sporadic ALS patients showed that most dysregulated genes were related to mitochondrial function. As a consequence, this result highlights mitochondrial participation in motor neuron degeneration and indicates that cell autonomous mechanisms could be associated with sporadic ALS (Alves et al., 2015).

### Modeling FTD using iPSCs

#### MAPT

From a pathological point of view, there are three main subtypes of FTD: FTD-TDP, FTD-FUS, and FTD-tau. Tau pathology is also a hallmark of Alzheimer's disease and progressive supranuclear palsy (Xia and Dickerson, 2017). FTD-tau is caused by mutations in the *MAPT* gene which encodes the tau protein (Rovelet-Lecrux et al., 2010; Ling et al., 2013). MAPT transcripts are diverse and widely expressed in the nervous system. The expression level of these transcripts depends on the neuronal maturation stage and the neuronal subtype. Tau is a microtubule-associated protein which is highly expressed in neurons (Rovelet-Lecrux et al., 2010).

Fong and colleagues generated iPSC from an individual carrying a heterozygous A152T mutation in tau (Fong et al., 2013). By using zinc-finger nuclease-mediated gene editing, they developed two isogenic iPSC lines: one with the point mutation corrected, and one with a homozygous point mutation. These iPSCs were successfully differentiated into neurons which was confirmed by a “microtubule-associated protein 2” (MAP2) staining (Fong et al., 2013). The survival of these neurons was affected by the point mutation (Fong et al., 2013). Axonal degeneration and caspase-cleaved tau fragmentation increased in severity from heterozygous to homozygous mutant lines, while corrected isogenic control lines remained normal (Fong et al., 2013). Furthermore, the amount of phospho-tau-positive neurons also increased in a dose-dependent manner. Taken together, this iPSC-derived model mimicked the tau pathology *in vitro*, confirmed the causal link between the tau mutation and tau pathology and helped to elucidate some of the key underlying pathogenic mechanisms.

#### GRN

The *GRN* gene encodes a progranulin precursor protein which is cleaved into different Granulins (Grns) that are glycosylated and secreted. Progranulin as well as GrnE have neurotrophic actions both *in vitro* and *in vivo* (Bhandari and Bateman, 1992; Van Damme et al., 2008). Autosomal dominant mutations in the *GRN* gene have been implicated in up to 25% of familial FTD cases and these mutations seem to cause the disease due to haploinsufficiency (Guven et al., 2016; Rainero et al., 2017).

To investigate the pathogenic mechanism of *GRN* in FTD, two research group created iPSCs from FTD patients with different mutations in *GRN*. Almeida and colleagues differentiated iPSCs containing a non-sense mutated *GRN* (S116X) into neurons and microglia (Almeida et al., 2012). The mixed postmitotic neurons expressed MAP2, vesicular glutamate transporter 1, glutamate decarboxylase and tyrosine hydroxylase. Compared to controls, no differences were observed in the neuronal differentiation of mutant cells (Almeida et al., 2012). Grns mRNA levels were significantly decreased in fibroblasts, iPSCs and iPSC-derived neurons from the GRN^S116X^ FTD patient compared to normal control and sporadic FTD patients (Almeida et al., 2012). In addition, GRN^S116X^ neurons were more sensitive to specific protein kinase inhibitors which indicates that the PI3K/Akt and MEK/MAPK signaling pathways could be involved in the molecular pathogenesis of FTD. Neither mitochondrial nor oxidative stressors increased the sensitivity of FTD patient derived neurons, implicating that these two pathway were not affected by the GRN level (Almeida et al., 2012). Both neurons from *GRN*^*S*116*X*^ FTD patient and sporadic FTD patient showed vulnerability to ER stress in a GRN-independent way (Almeida et al., 2012).

We were involved in the creation of iPSCs from FTD patients carrying the *GRN*^*IVS1*+*5G*>*C, null*^ mutation and haploinsufficiency of *GRN* was observed in these FTD-iPSCs (Raitano et al., 2015). The iPSCs were further differentiated into cortical neurons, which are affected in FTD patients. Although neuroprogenitors could be generated without any problem, the generation of “COUP-TF-interacting protein 2” (CTIP2), forkhead box protein P2 or “T-box, brain, 1” (TBR1) positive cortical neurons was dramatically lower in FTD patient lines compared to lines from their normal family members. This was specific for cortical neurons as the FTD-iPSCs could be normally differentiated into motor neurons. After introduction of *GRN* cDNA into the AAVS1 locus of the FTD-iPSC line, the cortical neurogenesis defect was restored. RNA-seq data showed that genes related to the Wnt/β-catenin signaling pathway were higher expressed in FTD-iPSCs compared to a corrected control line or an ESC line (Raitano et al., 2015). After treatment with IWP2, an inhibitor of active β-catenin levels, more brain lipid-binding protein- and CTIP2-positive cells were generated from the FTD patient lines. All together, these results highlight the Wnt signaling pathway as a potential therapeutic target in FTD (Raitano et al., 2015).

#### C9ORF72

The first iPSCs from FTD patients with *C9ORF72* hexanucleotide repeats were created by Almeida and colleagues (Almeida et al., 2013). They developed several iPSC lines from two patients with *C9ORF72* repeats (G_4_C_2_ > 1,000 repeats) belonging to the same FTD family. Similar to the effect of other mutations causing ALS/FTD, the maturation process of neurons was not impaired. RNA foci were observed in fibroblasts, iPSCs, iPSC-derived neurons from the patient cell line, but not in healthy controls. RAN translational products were also found in the iPSCs-derived neurons from patients. Decreased cell viability and increased caspase-3 activation were observed in human neurons carrying G_4_C_2_ repeat expansions after treatment with two different autophagy inhibitors (Webster et al., 2016). Furthermore, p62 significantly accumulated in neurons with *C9ORF72* repeats, but not in neurons derived from control iPSCs of healthy persons or neurons derived from iPSCs of an FTD patient with a *GRN* mutation (Webster et al., 2016). These results strongly suggest an involvement of autophagy in *C9ORF72*-FTD.

## Current limitations to iPSC technology

**Figure 2:**
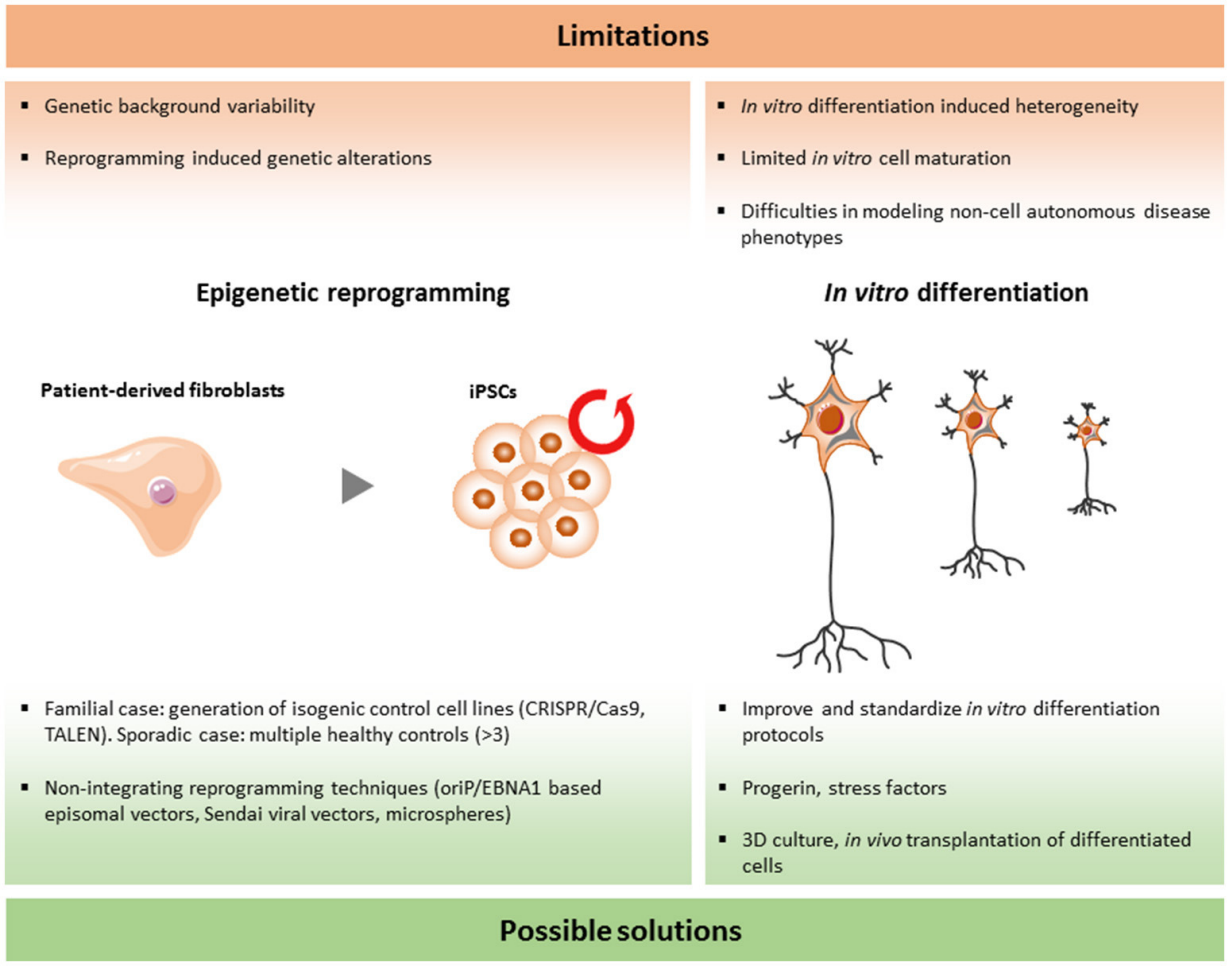
Limitations and current solutions to iPSC technology in ALS/FTD.

Although the iPSC technology has been used for modeling human diseases and drug screenings worldwide, there are still a number of limitations that should be solved. In the research area of ALS and FTD, more specific questions have to be answered in order to validate and improve this emerging tool (Figure 2).

### Dissimilarity among iPSC lines

As more and more iPSC lines are established and compared between different laboratories, large variations are discovered among different iPSC lines and, surprisingly, even between iPSC lines originating from the same donor sample. As a consequence, it is extremely important to standardize the different procedures involved in reprogramming in order to minimize these differences.

#### Setting back the clock: the reprogramming process

The initial work of Shinya Yamanaka in 2007 drew global interest to iPSC disease modeling. In their first description of the reprogramming method of somatic cells, four transcription factors (Oct3/4, Sox2, Nanog, cMyc) were delivered to human fibroblasts by integrating retroviral vectors (Takahashi et al., 2007). This method is still widely used to develop iPSC lines. However, this will result in the random insertion of these transgenes into the genome. The exact effect of this random integration is unknown and genomic modifications could have an unexpected and unwanted impact on the iPSC lines. For instance, transcriptional changes of certain genes could interfere with the analysis of the phenotypes. To avoid this drawback, Cre/loxP recombination and piggyBac transposon systems have been proposed to remove the transgenes, but both of these methods are time consuming and have a low efficiency (Karow et al., 2011; Zhou and Zeng, 2013). The introduction of integration free vectors is a milestone to avoid this problem in the reprogramming process. The first integration free reprogramming method was the cloning of the reprogramming factors into oriP/EBNA1 based episomal vectors, which are gradually lost during cell proliferation (Yu et al., 2009). Another method is the use of inactivated Sendai viral vectors. These are RNA virus based vectors with a high transfection efficiency, without the capability to integrate into the genome and they are also easily removed after a few cell passages. Another concern which was raised from research with mouse iPSCs is that the selection of reprogramming factors can influence the differentiation potential of iPSCs (Buganim et al., 2014). The use of the standardized integration free “Sendai Reprogramming Kit” with well-characterized reprogramming factors and protocols may help to avoid these unwanted effects (Trott et al., 2017). In addition, a novel approach was proposed recently by using microspheres conjugated with reprogramming factors for delivery into human fibroblasts (Unciti-Broceta et al., 2015). If this is a success, the use of small molecules to reprogram could be the next important step.

#### Pluripotency

A critical step in the procedure to obtain iPSCs is the characterization of the pluripotency potential. The final goal of the reprogramming process is to obtain iPSCs that are comparable to ESCs with respect to their pluripotency. The traditional status quo for pluripotency characterization is a combination of gene and antigen expression analysis, morphology, and capacity to differentiate *in vivo*. With respect to this procedure, several questions could be raised. In addition, the random inactivation of one of the X chromosomes (lyonization) is also a factor that could cause variation amongst female iPSC lines (Tomoda et al., 2012; Dandulakis et al., 2016). This can change the gene expression of some neuronal genes located on the X chromosome.

Analysis of teratomas has always been the golden standard for the confirmation of pluripotency (Nelakanti et al., 2015). However, it has been reported that even partially reprogrammed colonies can form teratomas *in vivo* (Baker, 2012). Therefore, more landmarks have been proposed to identify a fully reprogrammed iPSC. In theory, and to avoid these problems, a whole genome transcription and methylation analysis could provide more reliable information. In practice, this technology is expensive and is not available to every lab. As a consequence, the ideal situation would be the establishment of an international consortium collecting and storing all fully characterized iPSC lines containing all possible information for each of these established iPSC lines.

#### Controls

For iPSC disease modeling, the ultimate identification of disease phenotypes largely depends on the availability of reliable controls. It was shown in numerous situations that genetic background could play an important role in the disease manifestation and disease progression (Turner et al., 2017). For ALS and FTD, some causative genes have already been identified, but even for these there are huge variations in the disease process (Régal et al., 2006). It is not clear what causes these differences. However, unknown environmental factors and genetic risk factors may play a role in these dissimilarities. In order to avoid the influence of this genetic background on the disease-related phenotypes, proper controls are crucial. For the familial cases, genome editting is a promising strategy to correct point mutations or to remove pathological repeats in iPSC lines in order to create isogenic controls. Zinc finger nucleases, TALENs, as well as the more recently discovered CRISPR guided Cas9 nuclease system are the widely used technologies to perform these modification (Carroll, 2011; Beurdeley et al., 2013; Ran et al., 2013). A method based on piggyBac transposons allows seamless genome modification in patient derived iPSCs (Xie et al., 2014). The isogenic control keeps exactly the same genetic background as the mutant iPSCs. As there is still the risk of inducing off-targets effects or unwanted mutations by using these gene engineering technologies, exon sequencing and the choice of unique target sequences is crucial to minimize these drawbacks (Cho et al., 2014). In conclusion, corrected isogenic lines can be considered as the best controls for the familial type of ALS/FTD patient lines.

### Differences between differentiation methods

Numerous neuronal subtypes exist and selective vulnerability of these different neurons to the disease process is typical for the various neurodegenerative disorders. Motor neurons are mainly degenerating in ALS, while 70% of the affected cells in FTD patients are pyramidal cortical neurons (Rowland and Shneider, 2001; Boxer and Miller, 2005; Ling et al., 2013). By using different combinations of small molecules, several methods were developed to differentiate iPSCs into motor neurons or cortical neurons, but with varying efficiencies.

#### Identity of motor neurons

According to developmental studies, motor neuron specification goes through multiple steps and results in different subtypes of motor neurons. The inner cell mass first differentiates into ectoderm. Later, the inhibition of TGF-β/Smad signaling in combination with the enhancement of FGF and Wnt signaling triggers the specification from ectoderm to neuroectoderm (Vallier et al., 2009; Davis-Dusenbery et al., 2014; Kiecker et al., 2016). In the next step, a retinoic acid (RA) gradient and the activity of FGFs and “growth differentiation factor 11” (Gdf11) promote the neural tube pattern which results in the initial status of the central nervous system (Davis-Dusenbery et al., 2014; Maury et al., 2014). By the different regulation of *CDX* and *HOX* gene expression, motor neuron can be further specified into different subtypes (Davis-Dusenbery et al., 2014; Kiecker et al., 2016). For spinal cord motor neuron specification, “sonic hedgehog” (Shh), “bone morphogenic protein” (BMP)/TGFβ signaling, and the floor plate in the neural tube function together to trigger this specific differentiation pattern (Ribes et al., 2010).

*In vitro* differentiation from ESC/iPSC to motor neurons is also based on this process by triggering the different gene expression in every stage of the differentiation. The best way to generate motor neurons *in vitro* is to use small chemicals with different dosages at specific intervals to mimic every step of specification that takes place during development. The first description of motor neuron differentiation from human ESCs was published in 2005 and was based on RA inducing neuronal rosette formation and Shh inducing spinal cord motor neuron specification (Shin et al., 2005). This protocol results in Hb9/Islet1 positive, functional motor neurons with about 20% efficiency. Moreover, it is a time consuming protocol which requires 33 days to achieve initial motor neurons and a much longer time in order to gain functional mature motor neurons (Shin et al., 2005). Subsequently, several modified protocols were optimized based on the original one (Sances et al., 2016). They commonly share three key phases: neuronal induction, motor neuron specification, and motor neuron maturation. SB-431542, LDN-193189, and dorsomorphin were used for inhibition of TGFβ/BMP signaling in order to trigger neuronal differentiation (Patani et al., 2011; Amoroso et al., 2013; Gouti et al., 2014; Kiskinis et al., 2014; Maury et al., 2014; Du et al., 2015). In addition, the FGF pathway and Wnt signaling were also used to promote this differentiation stage (Dimos et al., 2008; Hu et al., 2009; Patani et al., 2011; Gouti et al., 2014). Nestin, PAX6 and Sox1 expression are used to check the quality of the neuronal progenitors (Yuan et al., 2011; Tian et al., 2013; Li et al., 2017). RA is used to link the neuroprogenitor phase to the motor neuron specification phase and a combination of RA, Shh, purmorphamine or SAG were used to initiate the Shh signaling pathway which contributes to the ventralization (Lee et al., 2007; Dimos et al., 2008; Amoroso et al., 2013; Kiskinis et al., 2014; Maury et al., 2014; Qu et al., 2014). The motor neuron progenitors should be positively stained for Olig2 (Masahira et al., 2006; Li et al., 2008; Hu and Zhang, 2009; Lee et al., 2014). It was reported that γ-secretase inhibition can significantly increase the motor neuron production upon SHH pathway activation (Maury et al., 2014). The expression of Hb9 and Islet1 expression are used as the criteria for the success of the initial motor neuron differentiation (Davis-Dusenbery et al., 2014). For the final motor neuron maturation, neurotrophic factors are applied to promote the formation of mature functional motor neurons which form functional synapses, that are electrophysiologically active and that can form neuromuscular junctions when co-cultured with myoblasts (Chen et al., 2014; Davis-Dusenbery et al., 2014; Sances et al., 2016).

As more and more protocols are reported, it is crucial that the criteria used to obtain and to characterize motor neurons are harmonized. This harmonization is also important for drug screening and to increase the success of translation of the results to the clinic. This could also help to obtain more convincing and reproducible results, as some findings obtained with motor neurons derived from iPSCs couldn't be replicated in different labs. This could, at least partially, be due to the different protocols using different molecules at different concentrations. For instance, the level of antioxidants present in the culture media could affect ALS-related phenotypes, as oxidative stress seems to be involved in triggering the disease process (Barber et al., 2006; Mattson and Magnus, 2006; Niedzielska et al., 2016). In addition, the amount and/or type of neurotrophic factors could also affect the appearance of ALS-related phenotypes, as some neurotrophic factors could affect ALS (Henriques et al., 2010). This should also be taken into account. As indicated previously, checking specific motor neuron markers (Islet1, Hb9, …) in combination with functional analysis is crucial to identify motor neurons in culture (Davis-Dusenbery et al., 2014; Sances et al., 2016).

By definition, both upper and lower motor neurons are affected in ALS patients (Nijssen et al., 2017). Although most of the protocols are focused on making spinal cord motor neuron with a high efficiency, only one study optimized a protocol to induce around 52% of upper motor neurons, characterized by PHOX2B and TBX20 upregulation (Maury et al., 2014). This was achieved by using lower concentrations of RA and Wnt agonist (Maury et al., 2014). This opens new perspectives as it gives the opportunity to study different motor neuron types, which could also help to gain more insights into ALS.

Another important drawback of iPSC modeling is that the motor neurons are not in their natural environment. Even co-cultures of neurons with other cell types will never be able to model the complexity of the *in vivo* environment. One possible solution could be to inject iPSCs into the central nervous system in animal models to study their integration, survival, and behavior in the *in vivo* enviroment.

#### Reliability of cortical neuron cultures

During development, mammalian cortical neurons start specialization in the rostral dorsal part of the neural tube (Pierani and Wassef, 2009; O'Leary et al., 2013). The neuroepithelial cells near the ventricular zone (VZ) and subventricular zone (SVZ) develop into the pyramidal neurons, interneurons, and glial cells in the cortex (Noctor et al., 2004). The early postmitotic neurons subsequently migrate away to form the cortical plate (CP) and then separate into a marginal zone (MZ, Layer I) and a deep subplate (SP) (Noctor et al., 2004). Cajal-Retzius cell are developed from the layer I (Noctor et al., 2004). Other specific cortical layers (II-VI) are formed from the CP (Noctor et al., 2004). The SP is formed by migrating through the intermediate zone (IZ), which is a layer that finally contains the axonal tracts of the cortex. At the same time, the SVZ appears between the VZ and the IZ (Noctor et al., 2004; Clinton et al., 2014). The SVZ mainly produces glia (Noctor et al., 2004; Clinton et al., 2014). The glia function as scaffolds to support and direct the migration of new neurons from the VZ to the CP (Noctor et al., 2004; Clinton et al., 2014). Maturing neuronal axons migrate out toward their targets and form functional synapses (Noctor et al., 2004).

The cortical neuron differentiation *in vitro* seems to be also very complicated. There are two main reasons for this. First, the cortex contains plenty of neuronal subtypes, which work together in a very complex way. Second, the architecture of the different cortical layers is very difficult to achieve *in vitro*.

In order to address the differentiation in the different cortical subtypes, a protocol was developed without the addition of morphogens (Espuny-Camacho et al., 2013). Only the BMP inhibitor, Noggin, was used to increase the neuronal differentiation from human iPSCs (Espuny-Camacho et al., 2013). Furthermore, pyramidal neurons from different layers were obtained at different time points (Espuny-Camacho et al., 2013). Pax6 and Otx1/2 were expressed after 10–19 days of differentiation, which resembles early dorsal forebrain (Espuny-Camacho et al., 2013). The first neurons are positive for TBR1, calretinin, and reelin which are the markers for Cajal-Retzius neurons (Espuny-Camacho et al., 2013). Around 24–28 days, FOXP2 positive and TBR1/CTIP2 positive neurons of layer VI started to appear. At 37 days, CTIP2 positive neurons were present (Espuny-Camacho et al., 2013). From 40 days on, the TBR1-positive neurons were increasing, while calretinin positive neurons decreased (Espuny-Camacho et al., 2013). This resembles the TBR1+/calretinin- deep layer neurons (VI and V) (Espuny-Camacho et al., 2013). At the last time point (61–72 days), STATB2 positive neurons resembling the layer V and upper layer appeared in culture. In addition, CUX1/BRN2 positive neurons of the upper layer appeared at the end (Espuny-Camacho et al., 2013). At the same time, deep layer markers were downregulated. Electrophysiological properties were confirmed by patch clamp experiments. After transplantation into mouse brain, the *in vitro* derived cortical neurons could integrate properly and formed axonal projections and dendritic patterns (Espuny-Camacho et al., 2013). Another study reported the differentiation of cortical neurons by using different morphogens in a 2D system (Shi et al., 2012). In this 2D model, neuronal subtypes that belong to different cortical layers appeared at staggered time points, much like during development, and integrated amongst each other.

Another major challenge is to mimic the complex structure of the cortex *in vitro*. Some initial spatial patterns are observed in 2D culture. For instance, neuronal progenitors form rosettes which have typical apicobasal polarity and interkinetic nuclear migration (Espuny-Camacho et al., 2013). These features are typical in the VZ during cortical development (Eom et al., 2013). However, long term 3D cultures of hESC/iPSC derived neocortex could solve, at least partially, the problem of the complex cortical organization (Lancaster and Knoblich, 2014; Zhang Z.-N. et al., 2016). These 3D cultures started from hESC/iPSC (Lancaster and Knoblich, 2014). Wnt inhibitor and TGFβ inhibitor combined with low cell adhesion culture plate were used to trigger cortical generation during day 0 to day 18 (Lancaster and Knoblich, 2014). From day 18 onwards, further long term cortical neuroepithelial cultures were started by controlling the environment (proper substrate and 40%O_2_/5%CO_2_; Lancaster and Knoblich, 2014). These cortical neuroepithelial cells could be kept in culture for more than 13 weeks and formed multilayer structures including three neuronal zones (SP, CP, and Cajal-Retzius cell zones) and three progenitor zones (VZ, SVZ, and IZ), which were similarly organized as in the human fetal cortex (Lancaster and Knoblich, 2014). Although it is a complicated protocol, this breakthrough will certainly contribute to a better modeling of FTD *in vitro* and has the potential to be used for future drug testing.

### Relevance of phenotypes to ALS/FTD

#### Aging

Both ALS and FTD are age-related neurodegenerative diseases with symptoms occurring late in life. As a consequence, the question arises whether relevant phenotypes can be observed in cultured motor neurons that can be kept in culture for several weeks or at most a few months. When ALS/FTD patient are sick, their neurons have already suffered the consequence of the disease process for many years. In contrast, the iPSC-derived neurons are much younger and could more closely resemble the fetal stages, rather than an adult stage. In the best case, these young neurons could mimic the presymptomatic phase of ALS/FTD (Inoue, 2010). Despite the fact that proteomic data indicate that there are clear resemblances between motor neurons derived from endogenous spinal motor neurons (Toma et al., 2015), it is reasonable to expect that aging could play a crucial role in the development of the disease-related phenotypes. To properly mimic late stages of ALS/FTD *in vitro*, inducing aging and setting landmarks of aging could be necessary. Nine features of mammalian aging are proposed as typical hallmarks, including genomic instability, telomere attrition, epigenetic alterations, loss of proteostasis, deregulated nutrient sensing, mitochondrial dysfunction, cellular senescence, stem cell exhaustion, and altered intercellular communication (López-Otín et al., 2013). One approach of inducing aging in iPSC-derived neurons is to use progerin (Miller et al., 2013). Progerin is an aberrant form of the nuclear architectural protein lamin A and can cause the premature aging disease Hutchinson-Gilford Progeria via inducing DNA damage (Musich and Zou, 2011; Miller et al., 2013). It is also observed in physiological aging. By treating neurons with progerin for a few days, multiple aging-related characteristics were observed including genomic instability, telomere attrition, and reactive oxygen species (Miller et al., 2013). Although this study only tested this approach in dopaminergic neurons, it is worth attempting this strategy in other neuronal types.

Another recent approach is to directly convert fibroblasts into neurons by transfecting transcription factors with a cocktail of small molecules (Hu et al., 2015; Liu et al., 2016). These directly induced neurons, also called iNeurons, show an age-specific transcriptional background and an age-related reduction of the nuclear transporter RanBP17 (Mertens et al., 2015; Gopalakrishnan et al., 2017). These iNeurons could present with more relevant phenotypes as these induced neurons did not undergo a complete reprogramming which might repair the macromolecular damage which could contribute to ALS/FTD related phenotypes (Mertens et al., 2015; Gopalakrishnan et al., 2017). One disadvantage of these iNeurons is that the fibroblasts used as starting material are not an unlimited resource of cells.

#### Environmental factors

Apart from aging, environmental factors could contribute to ALS/FTD, although convincing evidence for this is still scarce (Ingre et al., 2015). These environmental factors could eventually include malnutrition, hypoxia and psychological stress (Ingre et al., 2015). All these factors could lead to oxidative stress. Clinically, oxidative stress biomarkers in cerebrospinal fluid, plasma, and urine are elevated in ALS/FTD patients, suggesting that oxidative stress could play a pivotal role in motor neuron or cortical neuron degeneration (D'Amico et al., 2013; Mao, 2013; Turner et al., 2013). Moreover, the recently approved drug, edaravone, is thought to exert its therapeutic effect by counteracting oxidative stress (Hardiman and van den Berg, 2017). In addition, oxidative stress is also a major event during aging (López-Otín et al., 2013). Induction of oxidative stress *in vitro* could be helpful to model ALS/FTD associated phenotypes. Arsenite treatment and hydrogen peroxide are two examples to induce stress (Henkler et al., 2010). Stress granules and FUS/TDP-43 protein aggregations were reported to appear after adding oxidative stress (Dewey et al., 2012; Carrì et al., 2015; Lenzi et al., 2015). In addition, shortage of certain growth factors in ALS (e.g., VEGF, BDNF, GDNF, CNTF, …) were observed and could contribute to ALS disease progression *in vivo* (Bogaert et al., 2010; Henriques et al., 2010). Therefore, removal of growth factors can serve as a strategy to induce phenotypes in *in vivo* models. Moreover, based on the discovery of physiological changes in ALS iPSC models (Wainger et al., 2014; Devlin et al., 2015; Naujock et al., 2016), addition of excitotoxins could also be a method to induce phenotypes.

## Conclusions

In this review, we systematically summarized the published iPSC models of ALS/FTD, along with their advancements in our knowledge of these complex diseases, but as well as their current drawbacks and discrepancies. Indeed, there are many advantages to modeling ALS/FTD using iPSCs, however, one always has to keep in mind that this is just another model for ALS/FTD. It remains an *in vitro* model with all the disadvantages that come with it. Neuronal cultures could provide results that might not be relevant to the *in vivo* disease process. As a consequence, translating results from iPSC-related *in vitro* work into clinical trials without prior confirmation in additional models or validation in patient material should be avoided.

## Author contributions

WG contributed to the writing and organization of the whole manuscript. LF contributed to the *C9ORF72* part and the figures of this review. RP contributed to the general improvement of the manuscript. LVDB contributed to the organization and quality control of this manuscript.

### Conflict of interest statement

The authors declare that the research was conducted in the absence of any commercial or financial relationships that could be construed as a potential conflict of interest.
